# Sporotrichosis Caused by *Sporothrix mexicana*, Portugal

**DOI:** 10.3201/eid1710.110737

**Published:** 2011-10

**Authors:** Nicolina Marques Dias, Manoel Marques Evangelista Oliveira, Manuel Azevedo Portela, Cledir Santos, Rosely Maria Zancope-Oliveira, Nelson Lima

**Affiliations:** Universidade do Minho, Braga, Portugal (N.M. Dias, C. Santos, N. Lima);; Centro de Investigação em Tecnologias da Saúde, Gandra-Paredes, Portugal (N.M. Dias, M.A. Portela);; Fundação Oswaldo Cruz, Rio de Janeiro, Brazil (M.M.E. Oliveira, R.M. Zancope-Oliveira)

**Keywords:** fungi, Sporothrix, sporotrichosis, human infection, zoonoses, letter

**To the Editor:** Sporotrichosis is a subcutaneous fungal infection present worldwide that is caused by traumatic inoculation or inhalation of spores of the dimorphic fungus *Sporothrix schenckii* complex (*1**–**3*). However, molecular studies have shown that the *S. schenckii* complex constitutes several cryptic infectious species (i.e., *S. albicans, S. brasiliensis, S. globosa, S. luriei, S. mexicana*, and *S. schenckii*). Marimon et al. (*4*) demonstrated 3 major clades grouped into 6 putative phylogenetic species. The natural habitats of these species are soil and plants. The species showed distinct pathologic behavior, antifungal responses, and phenotypes, which suggests that optimal clinical treatment may depend on the taxon involved in the sporotrichosis (*1*). Human infections have been reported primarily from the Americas, including Latin America (*3**,**5*). Asia (e.g., Malaysia, India, Japan), Africa, and Australia are also regions where infections are endemic (*6*). Although infections are rare in Europe, a case of human infection (*7*) and a case of an animal infection (*8*) have been described in southern Italy. We report a case of human sporotrichosis in which *S. mexicana* was isolated from a patient in Portugal.

A 34-year-old man sought care at a podiatry clinic in Vila Nova de Famalicão, Portugal, in 2009 for multiple polymorphous eruptions and ulcers on both feet. There was no obvious cause of the disease. Although the patient had traveled to Malaysia in 2003 and had worn open footwear every day, he did not recall receiving a skin wound. In 2004 in Portugal, subcutaneous nodules appeared in both feet, became ulcerated, and spontaneously healed. By 2005, more severe lesions had appeared and became a chronic infection in both feet and lower limbs. The symptoms were diagnosed erroneously as dyshidrotic eczema, and treatment with topical corticosteroids was unsuccessful.

Several skin fragments of the lesions were submitted for mycological assessment. Fungi were not found on potassium hydroxide slides of all samples. Filamentous fungal colonies were observed after 7 days of culture on Sabouraud dextrose agar slopes at 25°C. The fungus had hyaline septate hyphae, with hyaline and dematiaceous conidia compatible with *Sporothrix* spp. The isolate was accessed and preserved in the Micoteca da Universidade do Minho (MUM, Braga, Portugal) fungal culture collection and given the accession code MUM 11.02.

The macroscopic features and sporulation were analyzed by using cornmeal and potato dextrose agars. Clusters of intercalary or terminal conidia were formed by sympodial growth from differentiated conidiophores on both media. Sympodial conidia were hyaline or slightly pigmented. Sessile conidia were predominantly subglobose, obovoidal or ellipsoidal, and 3.35 ± 0.41 µm long by 2.30 ± 0.32 µm wide (Figure, panel A). A teleomorph was not observed. The colony diameter on potato dextrose agar after 21 days of incubation attained 40 mm at 30°C and 5 mm at 37°C. The yeast form was achieved by incubating the isolate on brain heart infusion agar on slants at 35°C ± 2°C for 7 days in a single subculture.

**Figure:**
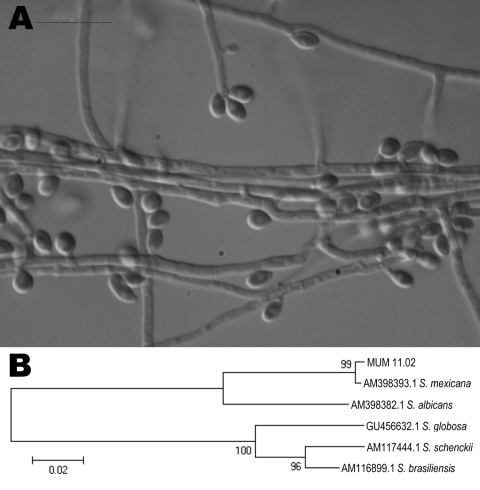
A) Photomicrograph of sympodial and sessile conidia of *Sporothrix mexicana* obtained by using a transmitted differential interference contrast microscope. The isolate was obtained from a patient in Portugal in 2009 and archived in the Micoteca da Universidade do Minho (MUM) under accession no. MUM 11.02. Scale bar = 10 μm. B) Neighbor-joining tree showing relatedness of MUM 11.02 isolate with other species of the *S. schenckii* complex. The percentage of replicate trees in which the associated taxon clustered in the bootstrap test (1,000 replicates) is shown next to the branches. All positions containing gaps and missing data were eliminated from the dataset (complete deletion option). There were 537 positions in the final dataset. Scale bar indicates nucleotide substitutions per site.

Dextrose, sucrose, and raffinose assimilation tests were performed in triplicate by using yeast nitrogen base medium. The strain assimilated dextrose, sucrose, and raffinose, showing phenotypic characteristics typical of *S. mexicana* and *S. schenckii* (*2*). In contrast, type reference strain *S. brasiliensis* CBS 120339 was included in the test, and it was able to assimilate only dextrose.

A presumptive identification based on phenotypic characteristics allowed us to classify this fungus as *S. mexicana*, although this species has an atypical morphologic profile. The diameter of colonies grown at 30°C and 37°C are smaller than those proposed by Marimon and collaborators but much closer to those of *S. schenckii* (*2*). These differences could be attributable to the intraspecific variation of this single isolate.

Genomic DNA was obtained from the yeast phase of *S. mexicana* MUM 11.02, and the partial sequencing of the nuclear calmodulin gene was based on the amplicon generated by PCR reaction by using CL1 and CL2A primers (*2**,**3*). Sequencing was performed at Fundação Oswaldo Cruz, Rio de Janeiro, Brazil. A BLAST analysis (www.ncbi.nlm.nih.gov/BLAST) comparing the sequence of the calmodulin gene with sequences AM398382, AM398393, AM117444, AM116899, and AM116908 in the GenBank database confirmed the identity of this isolate as *S. mexicana*. The MUM 11.02 isolate showed 99% similarity with the sequences of *S. mexicana* (i.e., GenBank accession no. AM398393) with high bootstrap support values (Figure, panel B). The calmodulin sequence of MUM 11.02 was deposited in GenBank as JF970258.

In vitro susceptibility tests with fluconazole, itraconazole, and terbinafine were performed by the microdilution method (*9*) and revealed MICs of 128 µg/mL, 32 µg/mL, and 0.5–1.0 µg/mL, respectively, which corresponds to the findings of Marimon et al. (*1*) for *S. mexicana*. Thus, *S. mexicana* is an emerging cause of human sporotrichosis.
